# Endangered plant species under differing anthropogenic interventions: how to preserve *Pterygopleurum neurophyllum* in Wondong wetland?

**DOI:** 10.7717/peerj.14050

**Published:** 2022-09-28

**Authors:** Seongjun Kim, Hwan-Joon Park, Chang Woo Lee, Nam Young Kim, Jung Eun Hwang, Byoung-Doo Lee, Hyeong Bin Park, Jiae An, JuHyoung Baek

**Affiliations:** Center for Endangered Species, National Institute of Ecology, Yeongyang, Gyeongbuk Province, Republic of Korea

**Keywords:** Conservation ecology, Influential factor, Soil compaction, Threatened species, Vegetation coverage, Wetland disturbance

## Abstract

Endangered wetland plants are important as the potential keystone species and mediators for plant-soil interactions. Establishing conservation strategies for endangered plants is also prioritized because of the elevating extinction risk by human-induced wetland disturbances. The present study examined the factors controlling the incidence of *Pterygopleurum neurophyllum*, the endangered wetland plant experiencing severe habitat loss throughout Northeast Asia. Here, *P. neurophyllum* populations and their surrounding environments were addressed in the last natural Korean habitat to assess the possible influential factors (vegetation coverage, species richness, exotic plant species, coarse rock content, soil bulk density, and soil electroconductivity and pH) under anthropogenic wetland interventions (with or without soil disturbance). Our results showed that *P. neurophyllum* occurred 6 out of 32 plots in the study area. All *P. neurophyllum* were found in *Miscanthus*-dominated area, but preferred microhabitats featuring reduced vegetation coverage, increased species richness, and undisturbed soils under vegetation removal. Multimodel inference also indicated that vegetation coverage (relative importance = 1.00) and coarse rock content (relative importance = 0.70) were the major influential factors for *P. neurophyllum* population size, and the surviving *P. neurophyllum* were strictly limited to where both of them were kept lowered. Furthermore, the wetland intervention with soil disturbance had a negative effect on *P. neurophyllum* by creating the rocky and compacted soil surface as a result of land reclamation treatments. Conversely, the wetland intervention without soil disturbance enhanced the *P. neurophyllum* incidence by decreasing vegetation coverage of the overcrowding competitive plants. Overall findings reflect that the strategies to counteract habitat loss and manage the overly dense competitive plants should be necessary for conserving *P. neurophyllum*, as well as other wetland plants threatened by the human-induced disturbances and excessive competition intensities.

## Introduction

Growing concerns are given to the risk of species extinction as well as the associated degradation of ecosystem functions and biodiversity. Protecting rare and endangered plants is important in terms of ecosystem functions since they can act as potential mediators of the plant-soil interaction and as keystone species in community structures (Dee et al., 2019). For example, several rare *Equisetum* plants can reallocate nutrients from the subsoil to the topsoil, and provide 29% of phosphorus input through foliage litterfall in Alaskan wetlands (Marsh et al., 2000). Because nutrient availability in grasslands is positively related to plant species richness in addition to legume presence (Reich et al., 2012), rare and endangered plants can also contribute to soil fertility and associated biomass production. Therefore, many researchers are tracking threatening factors, estimating the magnitude of extinction risk, predicting consequences, and establishing conservation strategies for endangered plant species around the world.

Several factors threaten endangered plants despite the importance of biodiversity protection. In particular, habitat loss and fragmentation are critical factors that can directly disturb reproduction, seed dispersal, and survival of endangered plants (Lander et al., 2019). The isolated endangered plants due to habitat fragmentation may also suffer from diminished pollinator activity, decelerated gene flow, and decreased genetic diversity (Honnay & Jacquemyn, 2007). In addition, the invasion of exotic species can elevate competition intensity because their fitness frequently outperforms native plants’ fitness, by which a danger of extinction can increase (Dueñas et al., 2018). Other biological factors such as outbreak of diseases and loss of symbiotic species can further magnify the possibility of extinction of endangered plants as well (Budiharta et al., 2011).

Endangered plants in wetland ecosystems have been more threatened recently because of the amplifying impacts of human activity. Artificial wetland modifications make landscape more terrestrial so that non-native, mesophilic species can eventually become invasive (Pattison, Vallejo-Marín & Willby, 2019). It further has a negative impact on taxonomic and functional diversity of ground arthropods beneficial for wetland plant community (Nanni et al., 2021). Mechanical disturbances by heavy machineries can also result in surface soil compaction that is harmful for wetland soil fertility, seed bank persistence, and rhizosphere composition (Wisheu & Keddy, 1991). Agricultural and industrial land uses may introduce contaminants into wetlands, which can also reduce soil fertility and nutrient cycling, and consequently cause a decrease in plant populations (Ljevnaić-Mašić et al., 2020). Moreover, excessive land reclamation can deteriorate hydrological regime of wetland ecosystems and destroy the entire wetland landscape (Im et al., 2017).

*Pterygopleurum neurophyllum* (Maxim.) Kitag is an endangered wetland plant, categorized as the perennial Apiaceae family. This species is historically distributed in Japan, southern China, and South Korean peninsula, and assessed as a legally threatened species throughout all the three countries: critically endangered (CR) class in South Korea and vulnerable (VU) class in China and Japan (National Institute of Biological Resources, 2021; Qin et al., 2017). However, *P. neurophyllum* is still impacted by agricultural, industrial, and recreational activities, and especially in South Korea, only one last natural habitat currently survives (National Institute of Biological Resources, 2021). Chinese and Japanese populations of *P. neurophyllum* are also facing to a similar situation, by which local extinction has been recorded from the urbanized and overexploited wetlands (Suzuki & Kokufuta, 1994; Yu et al., 2021). Such danger of extinction surrounding *P. neurophyllum* is unfortunately underrated due to the absence of information regarding population size and the relevant threatening factors, though previous researches investigated basic morphology (Rim & Chung, 1966), seed germination rate (Kwon, Kim & Kim, 2020), and coexisting flora composition and life form (Park et al., 2021). In this context, extra research efforts are required to elucidate the mechanisms hidden behind the population degradation, and to develop the detailed conservation strategies.

The present study aimed to clarify the factors influencing *P. neurophyllum* incidence and population size. The population surviving in the last South Korean habitat was specifically examined because it has been affected by agriculture, freshwater fishing, land reclamation, and river barrage construction so that it can represent the influential factors for *P. neurophyllum* under the anthropogenic wetland interventions (Oh et al., 2016; Son et al., 2002). The primary research questions were as follows: (1) Which factors play a major role in incidence and population size of *P. neurophyllum*? (2) Are there any relationships between the variations in the influential factors and anthropogenic wetland interventions? Through these questions, we sought potential threats for *P. neurophyllum* and developed several conservation guidelines for the endangered plants experiencing a similar danger from wetland interventions.

## Materials & Methods

### Study area

**Figure 1:**
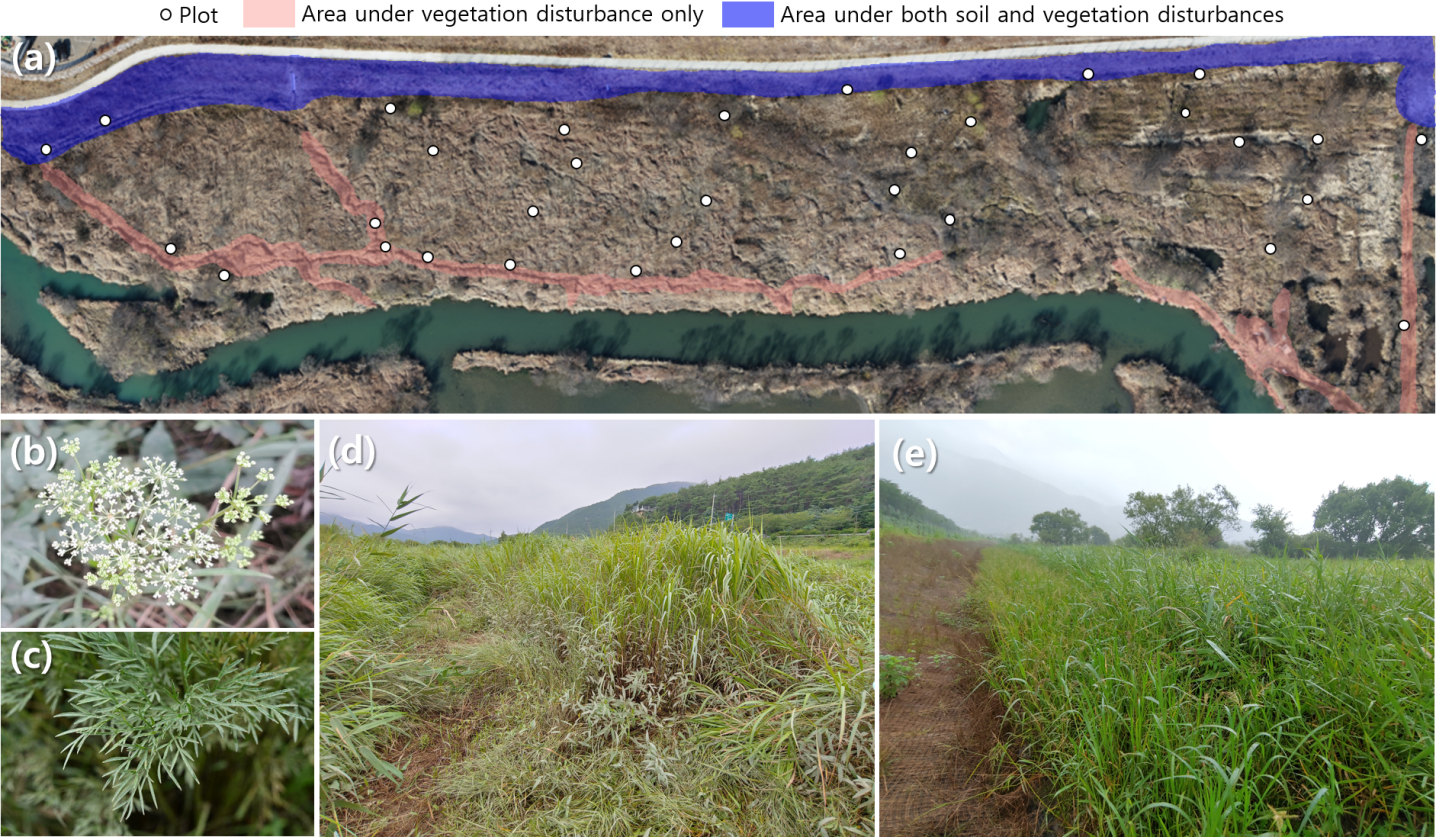
Photographs of study area and the target endangered species, *Pterygopleurum neurophyllum*. Location of plots (A), the target endangered species, *Pterygopleurum neurophyllum* (B, C), and examples of areas under vegetation disturbance only (D) and under both soil and vegetation disturbances (E).

The study area is located in Wondong wetland of Yangsan-si in South Korea (35°22′N, 128°54′E; Fig. 1), which is considered as the last natural habitat of *P. neurophyllum* in the country (National Institute of Biological Resources, 2021). The study area also provides habitats for another endangered wetland plant, *Viola raddeana* Regel (Park et al., 2021). This area is subjected to the warm-temperate climate with a hot, humid summer and a cold, dry winter, in addition to the concentrated rainfall event between July and September. Comprising the last 10 years, the average annual air temperature and precipitation are 14.9 °C and 1473, respectively. The Wondong wetland extends for approximately 57.3 ha, alongside the lower Nakdong River basin; thus, the study area has an altitude close to the sea level exhibiting minimum slope. Because of concentrated rainfall event during the summer, flooding seasonally occurs when Nakdong River overflows.

The study area has been affected by two types of anthropogenic wetland interventions for decades: wetland interventions without or with soil disturbance. The former results from continuous vegetation removal for the access to freshwater fishing; therefore, this area comprises the linear transects that contain a reduced vegetation coverage with marginal disturbance to the soil (area under vegetation disturbance only, Fig. 1A). On the other hand, the later originates from land reclamation along Nakdong River, which buries native wetland vegetation and creates bare soil patches. This area is, thus, characterized by reduced vegetation coverage, rocky and compacted soil surface, and terrestrial pioneer plant community such as *Setaria viridis* (L.) P. Beauv. (area under both soil and vegetation disturbances, Fig. 1A).

### Flora taxa and *P. neurophyllum* incidence investigations

A total of 32 plots were randomly established within the study area for flora monitoring in July and August 2021. This monitoring period was selected given that the flowering of *P. neurophyllum* occurred in the middle of the summer season. The size of each plot was 2 × 2 m, but it was extended to 5 × 5 m if the vegetation within the plot consisted of any mature tree species (*Salix* type vegetation, see below) considering the canopy width and height of arborescent communities. All plant taxa were listed (Table S1), and the exotic plant species were specified as well. The incidence and population size (number of individual) of *P. neurophyllum* were also checked. Species richness was calculated as the number of flora taxa in each plot.

Each plot was assigned to one of four vegetation types, namely *Setaria* (*n* = 3), *Miscanthus* (*n* = 16), *Phragmites* (*n* = 8), and *Salix* (*n* = 5). The *Setaria* type is dominated by *S. viridis*, and tends to occur around the terrestrial and compacted soil conditions (Fig. S1). The *Miscanthus* and *Phragmites* types are characterized as dense *Miscanthus sacchariflorus* (Maxim.) Hack. and *Phragmites australis* (Cav.) Trin. ex Steud. communities (Fig. S1). The overstory of the *Salix* type is covered by *Salix chaenomeloides* Kimura, *Salix pierotii* Miq., and *Salix koriyanagi* Kimura ex Goerz, while the understory is primarily vegetated by *M. sacchariflorus* and *P. australis* (Fig. S1).

The *Salix* type prefers the upper, drier wetland area compared to the *Miscanthus* and *Phragmites* types (Son et al., 2002). Both *Miscanthus* and *Phragmites* types occupy the lower, wetter conditions, but *M. sacchariflorus* more favors the micro-topography on sediment mounds than *P. australis* (Park et al., 2021). Furthermore, the *Miscanthus*, *Phragmites*, and *Salix* types have historically dominated Wondong wetland (Son et al., 2002), while the *Setaria* type is eventually introduced to the area under both soil and vegetation disturbances after land reclamation (Park et al., 2021). Accordingly, these four vegetation types are assumed to reflect both natural environments and recent impacts of anthropogenic interventions around the study area.

### Measurements for vegetation coverage and distance from disturbed area

Vegetation coverage was quantified in each plot using a drone (Phantom 4 pro V2.0, DJI, China) and the freeware software Imagej to estimate the potential competitive plant density (Banu, Borlea & Banu, 2017; Engelbrecht & Herz, 2001; Johnson, Manby & Devine, 2020). A photograph of the canopy was taken with a drone at 12 m height above the center of each plot at the same time of the flora investigation. Each photograph was clipped based on the edge of each plot, and processed following the vegetation coverage analysis protocol of Engelbrecht & Herz (2001); the type of each photograph changed from color to the grey, which led to the images with the black and white pixels representing vegetated and none-vegetated areas, respectively. Vegetation coverage of each plot was estimated by the percentage of the black pixels within the total number of pixels.

Distances from the area under vegetation disturbance only (D_V_) and under both soil and vegetation disturbances (D_SV_) were analyzed using drone mapping. A total of 547 photographs were taken at 40 m altitude across the study area using a grid mission procedure of Pix4Dcapture software (Pix4D, Prilly, Switzerland) with a drone. The photographs were integrated and mapped into a single image containing GPS data using Agisoft PhotoScan software (Agisoft, Saint Petersburg, Russia). D_V_ and D_SV_ were then estimated according to the displacement from the center of each plot to the line transect throughout the middle of the disturbed area on the mapped image data with QGIS software (QGIS Development Team 2021).

### Soil sampling and analyses

Coarse rock content, soil bulk density, soil pH, and soil electroconductivity were measured to detect potential variations in soils under the anthropogenic wetland interventions. In each plot, a soil core was collected at 0–10 cm depth using a cylindrical soil sampler (407 cm^3^). Soil electroconductivity was simultaneously measured in the field using a time-domain reflectometry sensor (TDR 150, Spectrum Technologies Inc., USA). Soil samples were air-dried and processed through a two mm sieve to separate coarse rocks (larger than two mm) from the fine soils (smaller than two mm). Coarse rocks and fine soils were oven-dried at 105 °C and then weighed to determine coarse rock content and soil bulk density. Coarse rock content was calculated by dividing the oven-dry mass of coarse rock to that of the total soil, and soil bulk density was based on the proportion of the oven-dry mass of the total soil relative to the volume of the soil sampler. Soil pH was measured by a 1:5 soil-to-water ratio with air-dried soil samples and a refillable electrode (Orion ROSS Ultra pH/ATC Triode; Thermo Scientific, Waltham, MA, USA).

### Statistical analyses

Plots were treated as the unit of replication for statistical analyses in the present study (*n* = 32, comprising three for *Setaria* type, 16 for *Miscanthus* type, eight for *Phragmites* type, and five for *Salix* type). Here, general linear model with Tukey’s HSD test was performed to assess the variation related to vegetation types (*P* < 0.05). Considering that all *P. neurophyllum* were detected in the *Miscanthus* type (Table 1), plots with *P. neurophyllum* (*n* = 6 out of 16) were analyzed separately from the other plots in the *Miscanthus* type (*n* = 10 out of 16) to find any differences related to *P. neurophyllum* incidence. Log transformation was applied to vegetation coverage, species richness, coarse rock content, soil electroconductivity, D_V_, and D_SV_ for normalization. Levene’s test was used to assess the homogeneity of variance considering the unbalanced experimental design (*P* > 0.05). Exotic plant incidence was analyzed as a binary variable (presence or absence) on the basis of Bernoulli distribution. For vegetation coverage, species richness, and exotic plant incidence, plot size was used as a random factor because the *Salix* type had larger plot size than the other types. These analyses were carried out with ggpubr and lme4 packages in R 4.2.1. software (R Core Team, 2022).

**Table 1:** Number of flora taxa and plots with exotic plant species and *Pterygopleurum neurophyllum* in each vegetation type.

Vegetation type	Number of plot	Order	Family	Genus	Taxa^a^	Number of plot with exotic plants	Number of plot with *P. neurophyllum*
*Setaria*	3	14	16	29	31	3	0
*Miscanthus*	16	15	16	28	34	2	6
*Phragmites*	8	9	10	15	20	1	0
*Salix*	5	10	12	16	21	2	0
All types	32	19	23	48	61	8	6

**Notes.**

a Including species, subspecies, and variety.

Permutational multivariate analysis of variance with post-hoc Bonferroni test was conducted with 9999 permutations to summarize the multivariate difference across D_V_, D_SV_, vegetation coverage, species richness, exotic plant incidence, coarse rock content, soil bulk density, soil electroconductivity, and soil pH (*P* < 0.05). This analysis was based on Bray-Curtis dissimilarity, for which input data were square root transformed. Given that our experimental design was unbalanced, permutational analysis of multivariate dispersion with 9999 permutations was accompanied to confirm the homogeneity of multivariate variance (*P* > 0.05). Permutational multivariate analysis of variance and permutational analysis of multivariate dispersion were performed with vegan and pairwiseAdonis packages of R 4.2.1. software.

Multimodel inference was adopted to elucidate major influential factors for *P. neurophyllum* population size. A series of models were created using all possible combinations of explanatory variables, and ordered in accordance with corrected Akaike information criterion (AIC_C_). Then, candidate models were selected until the cumulative Akaike weight exceeds 0.95, and used to calculate model-averaged coefficients (Symonds & Moussalli, 2011). Relative importance was estimated by summing Akaike weights of candidate models that include each explanatory variable, and significance of the full model was tested prior to multimodel inference (Symonds & Moussalli, 2011). Square root transformation was implemented for input data of multimodel inference. Simple linear and multiple regressions were also used to reveal any notable pairwise relationships between the variables (*P* < 0.05). Multimodel inference and regression tests were performed with MuMIn and berryFunctions packages of R 4.2.1. software (R Core Team, 2022).

## Results

### Flora taxa and *P. neurophyllum* incidence

A total of 61 flora taxa were detected in the study area (19 orders, 23 families, 48 genus, 57 species, one subspecies, and three varieties). Asteraceae (nine taxa), Poaceae (eight taxa), Polygonaceae (seven taxa), and Cyperaceae (six taxa) plants were prevalent in the study area and accounted for the half of the detected flora taxa. Monocotyledons and dicotyledons consisted of 42 and 19 flora taxa, while pteridophytes and gymnosperms were undetected in the study area. When each vegetation type was separated, the *Setaria*, *Miscanthus*, *Phragmites*, and *Salix* types included 31, 34, 20, and 21 flora taxa, respectively (Table 1). List of the flora taxa is available at Table S1.

The target endangered plant, *P. neurophyllum* was observed only in the *Miscanthus* type, but not in the other vegetation types (Table 1). Out of 16 plots belonging to the *Miscanthus* type, *P. neurophyllum* was found in 6 plots, which were located near the area under vegetation disturbance only. As a result, the plots with *P. neurophyllum* generally featured a less dense canopy relative to the plots without *P. neurophyllum*. Each population of *P. neurophyllum* included one to 17 individuals, and 36 *P. neurophyllum* individuals were found in total.

Ten exotic plant species were identified in the study area (*Ambrosia artemisiifolia* L., *Ambrosia trifida* L., *Conyza canadensis* (L.) Cronquist, *Conyza sumatrensis* (Retz.) E. Walker, *Cosmos bipinnatus* Cav., *Xanthium orientale* L., *Amaranthus patulus* Bertol., *Chenopodium album* L., *Amorpha fruticosa* L., and *Rumex nipponicus* Franch. & Sav.) (Table S1). The *Setaria*, *Miscanthus, Phragmites*, and *Salix* types contained eight, three, two, and two exotic plant species, respectively. All three exotic plant species of the *Miscanthus* type were found in the plots with *P. neurophyllum*, whereas there was no exotic plant in the *Miscanthus* type without *P. neurophyllum*. The *Setaria* type had the highest exotic plant incidence ( *P* < 0.005), and all plots belonging to the *Setaria* type included exotic plant species (Table 1).

### Variations related to vegetation type and *P. neurophyllum* incidence

Permutational analysis of multivariate dispersion confirmed the homogeneity of multivariate variance among D_V_, D_SV_, vegetation coverage, species richness, exotic plant incidence, coarse rock content, soil bulk density, soil electroconductivity, and soil pH (*P* > 0.05, Table 2), despite the unbalanced number of replication. Permutational multivariate analysis of variance and post-hoc test showed that the *Miscanthus* type with *P. neurophyllum* significantly differed from the *Miscanthus* without *P. neurophyllum* (*F* = 13.26, *P* = 0.01) and the *Phragmites* type (*F* = 11.44, *P* = 0.02). It suggests that the environmental conditions of the area with *P. neurophyllum* were possibly distinguishable from those of the area without *P. neurophyllum*, even when they shared the same vegetation type.

**Table 2:** Summary of permutational multivariate analysis of variance (PERMANOVA) and permutational analysis of multivariate dispersion (PERMDISP) on distance from area under vegetation disturbance only, distance from area under both soil and vegetation disturbances, vegetation coverage, species richness, exotic plant incidence, coarse rock content, soil bulk density, soil electroconductivity, and soil pH according to vegetation types (*Setaria*, *Miscanthus*, *Phragmites*, or *Salix*) and *Pterygopleurum neurophyllum* incidence (with or without *P. neurophyllum*) (permutation = 9999).

PERMANOVA results					PERMDISP results
	Degree of freedom	Sum of squares	*F* value	*P* value^*^	
Vegetation type	3	0.04	5.96	<0.005^*^	*P* = 0.79
*P. neurophyllum* incidence	1	0.01	3.26	0.08	*P* = 0.10
Residuals	27	0.06			

Post-hoc tests	
Compared groups	*P* value^a^
*Miscanthus* with *P. neurophyllum* vs *Miscanthus* without *P. neurophyllum*	0.01^*^
*Miscanthus* with *P. neurophyllum* vs *Phragmites*	0.02^*^
*Miscanthus* with *P. neurophyllum* vs *Salix*	0.10
*Miscanthus* with *P. neurophyllum* vs *Setaria*	0.08
*Setaria* vs *Miscanthus* without *P. neurophyllum*	0.05^*^
*Setaria* vs *Phragmites*	0.07
*Setaria* vs *Salix*	0.39
*Miscanthus* without *P. neurophyllum* vs *Phragmites*	0.45
*Miscanthus* without *P. neurophyllum* vs *Salix*	1.00
*Phragmites* vs *Salix*	1.00

**Notes.**

* Asterisk indicates statistical significance at *P* < 0.05.

a Adjusted by Bonferroni correction.

**Figure 2:**
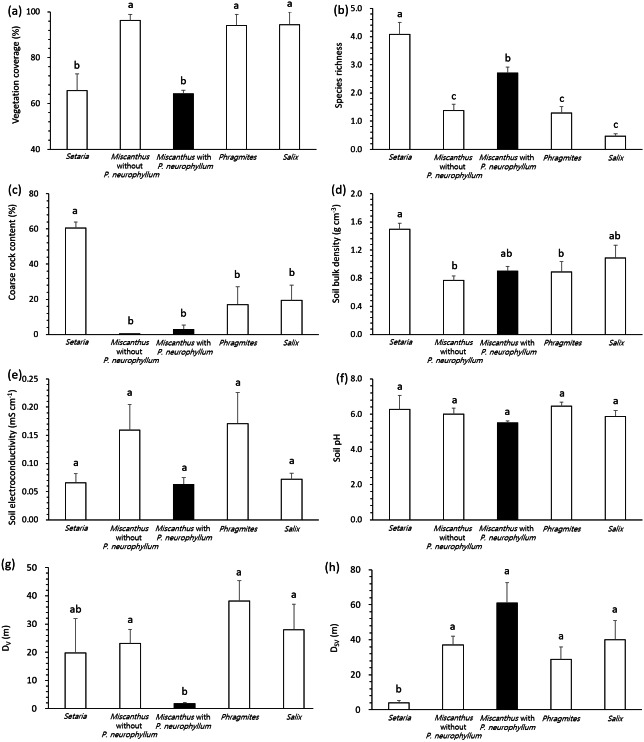
Vegetation coverage, species richness, coarse rock content, soil properties, and distances from disturbed areas among vegetation types and *Pterygopleurum neurophyllum* incidence. Comparisons of vegetation coverage (A), species richness (B), coarse rock content (C), soil bulk density (D), soil electroconductivity (E), soil pH (F), and distance from areas under vegetation disturbance (D_*V*_; G) and under both soil and vegetation disturbances (D_*S*_*V*; H) among vegetation types and *Pterygopleurum neurophyllum* incidence (*Setaria*: *n* = 3, *Miscanthus* without *P. neurophyllum*: *n* = 10, *Miscanthus* with *P. neurophyllum*: *n* = 6, *Phragmites*: *n* = 8, and *Salix*: *n* = 5). Vertical bars indicate standard errors. Values sharing same letters are not different at *P* < 0.05. Log-transformation is applied to vegetation coverage, species richness, coarse rock content, soil electroconductivity, D_*V*_, and D_*SV*_ for normalization. All variables comply with the assumption of homoscedasticity (*P* > 0.05).

All variables complied with the assumption of homoscedasticity (Levene’s test, *P* > 0.05), despite the unbalanced number of replication according to vegetation type and *P. neurophyllum* incidence. General linear model and pairwise comparisons demonstrated that the *Setaria* type and *Miscanthus* type with *P. neurophyllum* were lower in vegetation coverage (*F* = 13.10, *P* < 0.005, Fig. 2A) and higher in species richness (*F* = 17.74, *P* < 0.005, Fig. 2B) than the *Miscanthus* type without *P. neurophyllum*, *Phragmites* type, and *Salix* type. The *Setaria* type also showed the highest coarse rock content (*F* = 8.33, *P* < 0.005, Fig. 2C) and soil bulk density (*F* = 3.80, *P* = 0.01, Fig. 2D). Conversely, no significant difference was detected for soil electroconductivity (*F* = 1.42, *P* = 0.26, Fig. 2E) and soil pH (*F* = 1.18, *P* = 0.34, Fig. 2F). The *Miscanthus* type with *P. neurophyllum* had the lowest D_V_ (*F* = 4.13, *P* = 0.01, Fig. 2G), while the *Setaria* type exhibited the lowest D_SV_ (*F* = 4.28, *P* = 0.01, Fig. 2H).

### Influential factors for *P. neurophyllum* population size

**Figure 3:**
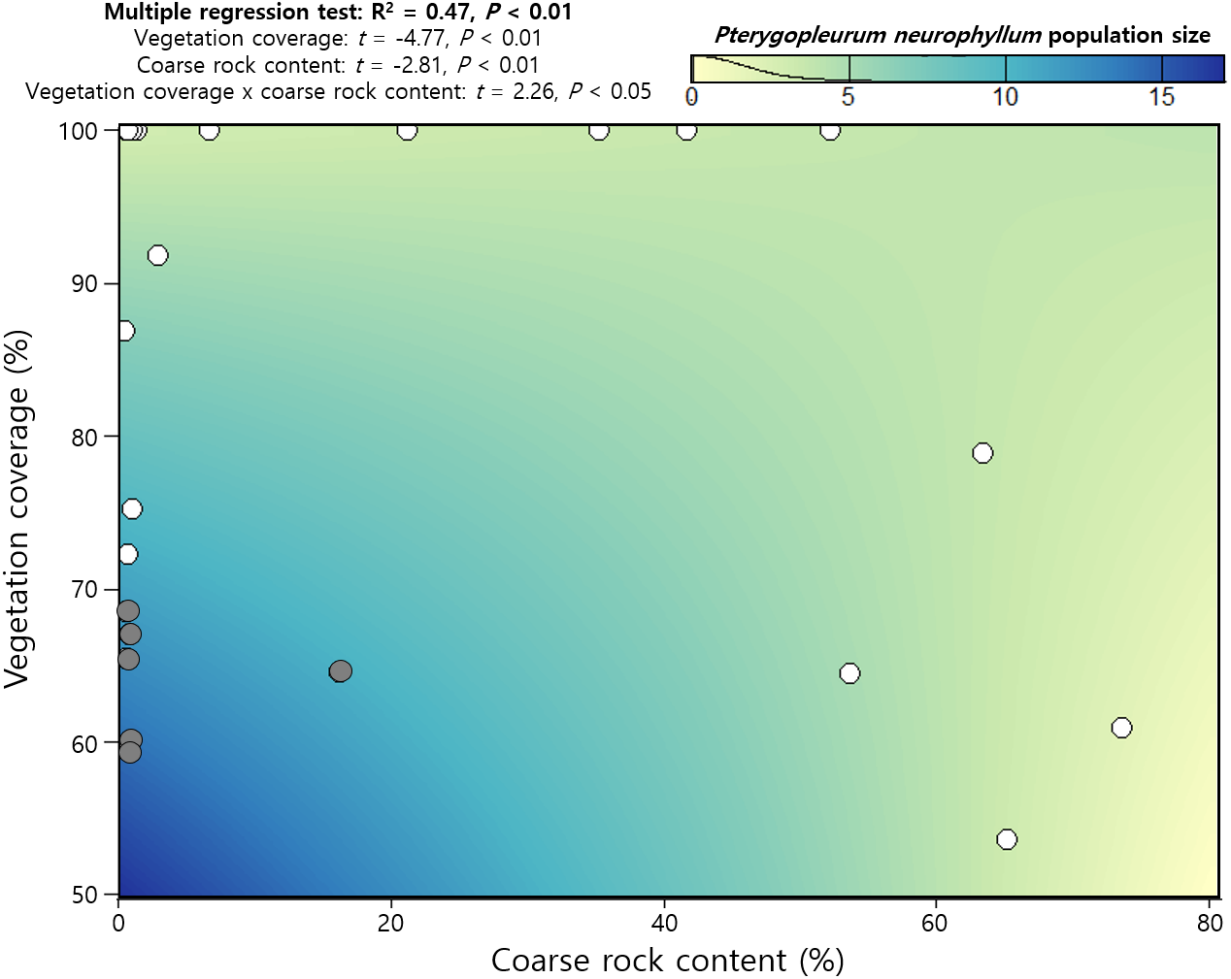
Regression results on *Pterygopleurum neurophyllum* population size using vegetation coverage, coarse rock content, and the interaction effect. Grey and white dots indicate the data from the plots with and without *P. neurophyllum*, respectively. The color gradient on the panel shows the estimated *P. neurophyllum* population size using the regression model (*n* = 32).

Multimodel inference selected 113 candidate models from 512 sets of created models, among which the first-ranked model included coarse rock content and vegetation coverage only (AIC_C_ = 74.3, Table 3). These two explanatory variables also had significant model-averaged coefficients and higher relative importance than D_V_, D_SV_, species richness, exotic plant incidence, soil bulk density, soil electroconductivity, and soil pH (Table 3). Notably, all candidate models contained vegetation coverage, and correspondingly, relative importance of vegetation coverage became 1.00. Multiple regression test found a positive interaction effect between coarse rock content and vegetation coverage, while each of them was negatively related to *P. neurophyllum* population size (Fig. 3). Incidence of *P. neurophyllum* was concentrated at the area that had relatively low vegetation coverage and coarse rock content; however, *P. neurophyllum* did not occur if either vegetation coverage or coarse rock content was high (Fig. 3).

**Table 3:** Results of multimodel inference to examine influential factors for *Pterygopleurum neurophyllum* population size (*n* = 32).

Full model including all variables		
Explanatory variables included^a^	R^2^	*P* value
Coarse rock content, D_SV_, D_V_, exotic plant incidence, soil bulk density, soil electroconductivity, soil pH, species richness, vegetation coverage	0.58	0.01

Candidate models^b^				
Rank	Explanatory variables included	AIC_C_^c^	ΔAIC_C_	Akaike weight
1	Coarse rock content, vegetation coverage	74.3	0.0	0.102
2	Coarse rock content, soil bulk density, vegetation coverage	75.4	1.1	0.058
3	Coarse rock content, exotic plant incidence, vegetation coverage	75.9	1.6	0.044
4	D_SV_, vegetation coverage	76.1	1.8	0.041
5	Coarse rock content, D_SV_, vegetation coverage	76.2	1.9	0.040
6	D_SV_, exotic plant incidence, vegetation coverage	76.4	2.1	0.036
7	Coarse rock content, soil electroconductivity, vegetation coverage	76.4	2.1	0.035
8	Coarse rock content, D_V_, vegetation coverage	77.0	2.7	0.026
9	Coarse rock content, species richness, vegetation coverage	77.1	2.8	0.025
10	Coarse rock content, soil pH, vegetation coverage	77.1	2.8	0.025
	⋮			
113	Coarse rock content, D_V_, Soil pH, Species richness, Vegetation coverage	83.3	9.0	<0.001

Model-averaged coefficients^d^					
Variable name	Parameter estimate	Standard error	*Z* value	*P* value^*^	Relative importance
Vegetation coverage	−0.718	0.176	4.08	<0.005^*^	1.00
Coarse rock content	−0.151	0.072	2.12	0.03^*^	0.70
D_SV_	0.124	0.094	1.32	0.19	0.38
Exotic plant incidence	−0.536	0.445	1.21	0.23	0.34
Soil bulk density	0.833	1.112	0.75	0.45	0.25
Soil electroconductivity	−0.481	1.282	0.38	0.71	0.18
D_V_	−0.014	0.088	0.16	0.87	0.18
Species richness	0.080	0.433	0.18	0.85	0.17
Soil pH	−0.003	0.981	0.00	0.99	0.17

**Notes.**

a D_V_: distance from area under vegetation disturbance only; D_SV_: distance from area under both soil and vegetation disturbances.

b Models selected until the cumulative Akaike weight exceeds 0.95.

c Corrected Akaike’s information criterion.

d Weighed averages over all 113 candidate models.

* Asterisk indicates statistical significance at *P* < 0.05.

**Figure 4:**
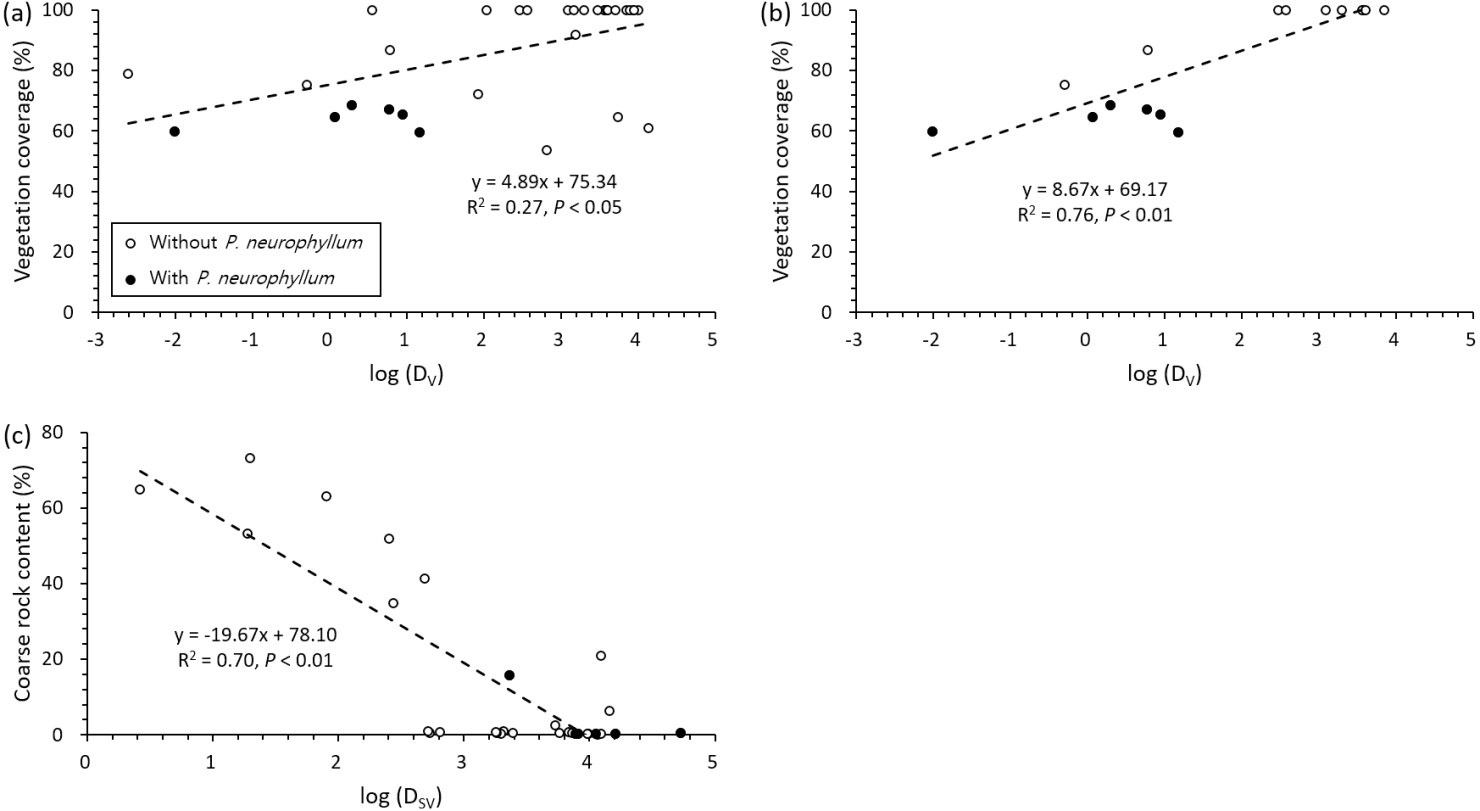
Regressions between vegetation coverage, coarse rock content, and distance from disturbed area. Pairwise relationships between vegetation coverage and distance from area under vegetation disturbance only (D_*V*_) across all vegetation type (A; *n* = 32) and within the *Miscanthus* type (B; *n* = 16) and between coarse rock content and distance from area under both soil and vegetation disturbances (D_*SV*_) across all vegetation type (C; *n* = 32). Black and white dots show the values from the plots with and without *Pterygopleurum neurophyllum*, respectively.

The two most influential factors (vegetation coverage and coarse rock content) for *P. neurophyllum* population size were significantly related to D_V_ and D_SV_ (Fig. 4). Vegetation coverage increased with D_V_, and *P. neurophyllum* occurred in the plots having low vegetation coverage and D_V_ (Fig. 4A). Such pattern was especially remarkable within the *Miscanthus* type (Fig. 4B). Meanwhile, coarse rock content decreased with D_SV_, and *P. neurophyllum* was found in the plots containing low coarse rock content and high D_SV_ (Fig. 4C). These results reflect differing effects between the reduced vegetation coverage without soil disturbance and the elevated coarse rock content under both soil and vegetation disturbances.

## Discussion

### *Pterygopleurum neurophyllum* incidence and influential factors

Our results show several distinctive characteristics of *P. neurophyllum* microhabitats, although all of them existed in the area dominated by *M. sacchariflorus*. Especially, the *Miscanthus* type with *P. neurophyllum* was clearly distinguishable from the *Miscanthus* type without *P. neurophyllum* by lower vegetation coverage, higher species richness and exotic plant incidence, and closer distance to the area under vegetation disturbance only. This pattern is inconsistent with previous reports on the degradation of endangered plant populations because of anthropogenic interventions and invasion of exotic species (Dueñas et al., 2018; Loope, Starr & Starr, 2004). Instead, the decreased *M. sacchariflorus* coverage following anthropogenic wetland interventions seemed to provide an opportunity for *P. neurophyllum* (and exotic plants) to survive, even though *M. sacchariflorus* is native to the study area.

It is notable that vegetation coverage was the most influential factor, negatively affecting *P. neurophyllum* population size in the *Miscanthus* type. In fact, *Miscanthus* plants are known by their invasive characteristics to the moist ecosystems, and could overcrowd both natural wetlands and abandoned agricultural lands (Schnitzler & Essl, 2015). Such introduction of *Miscanthus* plants might intensify the competition for light availability, and simplify plant community composition by shading out other coexisting plants and seed bank beneath the soil (Hager et al., 2015). Furthermore, *Miscanthus* plants primarily distribute their root-rhizome systems to the surface and subsurface soils for nutrient and water uptake (Neukirchen et al., 1999), which could exclude *P. neurophyllum* that has a similarly shallow rhizome depth (Park et al., 2021; Rim & Chung, 1966). These competition effects might hinder the growth and population development of *P. neurophyllum* beneath the dense *Miscanthus* canopy. In this context, our findings suggest that dominance by *M. sacchariflorus* could discourage the abundance of *P. neurophyllum* unless wetland interventions without soil disturbance decreased vegetation coverage.

If taking all vegetation types into account, vegetation coverage alone was not enough to explain overall variations in the incidence and population size of *P. neurophyllum*. Specifically, the *Setaria* type lacked *P. neurophyllum* incidence despite its low vegetation coverage as the *Miscanthus* type with *P. neurophyllum*. Mechanical soil disturbance might contribute to this pattern considering that the level of coarse rock content acted as the second influential factor for *P. neurophyllum* population size. Actually, the *Setaria* type was dominant across the area under both soil and vegetation disturbances, and featured higher coarse rock content than the *Miscanthus* type with *P. neurophyllum*. Rocky and compacted soil surface are considered to decline plant growth by reducing penetration ability of root systems, forcing the topsoil roots to become redistributed into the subsoil, and restricting nutrient uptake from the soil surface (Taylor & Brar, 1991; Unger & Kaspar, 1994). As *P. neurophyllum* is known to form a root-rhizome system mainly at the shallow soil depth (Rim & Chung, 1966; Park et al., 2021), the rocky topsoil condition might be crucial for nutrient acquisition of *P. neurophyllum* and consequently block the survival under anthropogenic soil disturbance.

### Differing effects of anthropogenic wetland interventions

Our results demonstrate that wetland interventions with soil disturbance could have a negative effect on *P. neurophyllum* population size by generating the rockier and compacted soil conditions. This negative response is not unanticipated because the excessive anthropogenic wetland interventions have been frequently pointed out as major threats of endangered plants (Ren et al., 2012). Excessive wetland interventions may distort the native plant community and surrounding environments by accelerating the invasion of competitive species, reorganizing the level of spatial heterogeneity, compacting soil profile, diminishing seed bank, reducing survival rate of remaining population, and altering hydrological regime and nutrient availability (Magnano et al., 2018; Mao et al., 2016; Schweiger et al., 2016; Wisheu & Keddy, 1991). When the magnitude of such interventions exceeds the ecosystem resilience, it may lead to the loss of regional wetland patches and habitat fragmentations (Chi et al., 2018; Deane et al., 2017). Although soil disturbance in the study area was not as severe to cause the entire habitat loss and extinction of *P. neurophyllum* at the current stage, its potential risk is undeniable for future fitness of the remaining population given the landscape alteration due to the overexploitation around the lower Nakdong River basin (Im et al., 2017; Oh et al., 2016).

The effects of wetland interventions without soil disturbance seemed to contrast with those of the interventions with soil disturbance. Reduced vegetation coverage owing to wetland interventions without soil disturbance had a positive effect on *P. neurophyllum* population size by lowering the magnitude of inter-species competition. In other words, the canopy without such vegetation disturbance might be too dense for *P. neurophyllum* to maintain their population in the study area. Obata et al. (2012) similarly predicted the improvement of endangered plant communities resulting from the restoration activity to remove invasive exotic plants in Watarase wetland, a historical *P. neurophyllum* habitat. These findings also affirm that creating canopy gaps could reorganize the monotonous landscape and enhance plant diversity (Anderson & Leopold, 2002; Scanga & Leopold, 2012; Wisheu & Keddy, 1991).

A similar situation may occur in the habitats of other endangered wetland plants. For instance, *V. raddeana*, an endangered plant cohabiting with *P. neurophyllum* is threatened by anthropogenic habitat loss and climate change (Jeong et al., 2018); nevertheless, Sawada et al. (2010) observed that prescribed burning boosted the occurrence of *V. raddeana* seedlings by decreasing the dominance of competitive plants in a wet secondary grassland. In fact, anthropogenic interventions sometimes mimic the role of historical, natural disturbances to wetland biodiversity and rare plant populations when their intensity does not surpass the level of ecosystems’ resilience (Zhang et al., 2020; Wisheu & Keddy, 1991). These tendencies denote the importance to develop conservation strategies for the endangered plants that are experiencing strong inter-species competitions and anthropogenic wetland interventions.

### Conservation implications

Several implications can be cautiously suggested for *P. neurophyllum* conservation in accordance with the differing effects of wetland interventions. First and foremost, any extreme land reclamations should be excluded from the *P. neurophyllum* habitat. This is essential to prevent further progression of heavy soil disturbance and habitat loss for remaining *P. neurophyllum* populations. Second, partial reduction of competitive plants can be implemented to provide extra spaces for *P. neurophyllum* so that they can avoid intense resource competitions beneath the dense vegetation coverage. This treatment should involve neither the soil compaction by heavy machineries nor the incorporation of rocky materials to minimize soil disturbance. Since establishing canopy gaps could accelerate the unintended invasion of exotic plants as well (Brewer, 2010), such treatment on vegetation coverage should be followed by careful monitoring regarding the shift in plant community composition.

Direct reinforcement of *P. neurophyllum* population should also be addressed because small population size is generally unfavorable in terms of maintaining the safe sites for fundamental niches and minimum viable population (Zedler, 2000). Therefore, if necessary, conservation programs may consider the inclusion of artificial propagation and reintroduction into the natural habitat to help enlarge *P. neurophyllum* populations. Although several institutes have established ex-situ collections of *P. neurophyllum* and studied artificial seed germination (Kwon, Kim & Kim, 2020), sufficient number of individuals for large-scale restoration may not be available because of the extremely small wild population. In this regard, a small-scale experimental reintroduction would be an option to initiate the successive population reinforcement and clarify factors impacting the ecophysiology of planted *P. neurophyllum* more in detail.

Another point deserving consideration is the conflict around the study area between wetland protection and modification for agricultural, industrial, and recreational purposes. Similar conflicts also prevail across other wetlands alongside the lower Nakdong River basin (Im et al., 2017), which can be alternative sites for the experimental reintroduction of *P. neurophyllum*. This situation is hindering the feasibility of conservation activities, although South Korean government declares *P. neurophyllum* as one of the species requiring urgent restoration. Thus, policy makers and scientists should cooperate to pursue socioeconomic agreements regarding how to manage the remaining habitat for *P. neurophyllum*.

## Conclusions

The importance of endangered plants is undeniable in terms of biodiversity conservation and ecosystem restoration. In particular, preservation of endangered wetland plants is one of the top research priorities due to recent conflicts between wetland protection and overexploitation. The present study uncovers that the anthropogenic wetland interventions had differing effects on the last South Korean habitat for *P. neurophyllum*. This effect was closely related to the variability in vegetation coverage and coarse rock content according to the anthropogenic interventions. Importantly, rocky and compacted soil conditions owing to soil disturbance had a negative impact on *P. neurophyllum*, while decreased vegetation coverage without soil disturbance helped maintain *P. neurophyllum* population by reducing competition intensity under overcrowding *Miscanthus* plants. The overall findings highlight the necessity of conservation strategies not only to regulate wetland overexploitation and total habitat loss, but to provide additional opportunities for remaining *P. neurophyllum* to avoid excessive competition as a result of dominance by a single plant species. A similar approach may be applicable to conserving the wetland plants threatened by the effects of human activities and overly dense competitors.

##  Supplemental Information

10.7717/peerj.14050/supp-1 Supplemental Information 1 Photographs of each vegetation type, which were dominated by *Setaria viridis* (a), *Miscanthus sacchariflorus* (b), *Phragmites communis* (c), or *Salix* species (d) Photographs of each vegetation type, which were dominated by *Setaria viridis* (a), *Miscanthus sacchariflorus* (b), *Phragmites communis* (c), or *Salix* species (d).

10.7717/peerj.14050/supp-2 Supplemental Information 2 List of flora taxa detected in each vegetation type Table S1. List of flora taxa detected in each vegetation type (A: *Setaria* type; B: *Miscanthus* type; C: *Phragmites* type; D: *Salix* type). Asterisk indicates exotic plant species.

10.7717/peerj.14050/supp-3 Data S1 Raw data All plant species found in the study site, and primary data that are used for all the statistical analyses.

## Additional Information and Declarations

### Data Availability

The following information was supplied regarding data availability: The raw data are available in the Supplemental Files.
