# Supplementary figures and images for: Endangered plant species under differing anthropogenic interventions: how to preserve Pterygopleurum neurophyllum in Wondong wetland?
Source: PeerJ. 2022 Sep 28;10:e14050. doi: 10.7717/peerj.14050 (PMC9526420; doi:10.7717/peerj.14050)

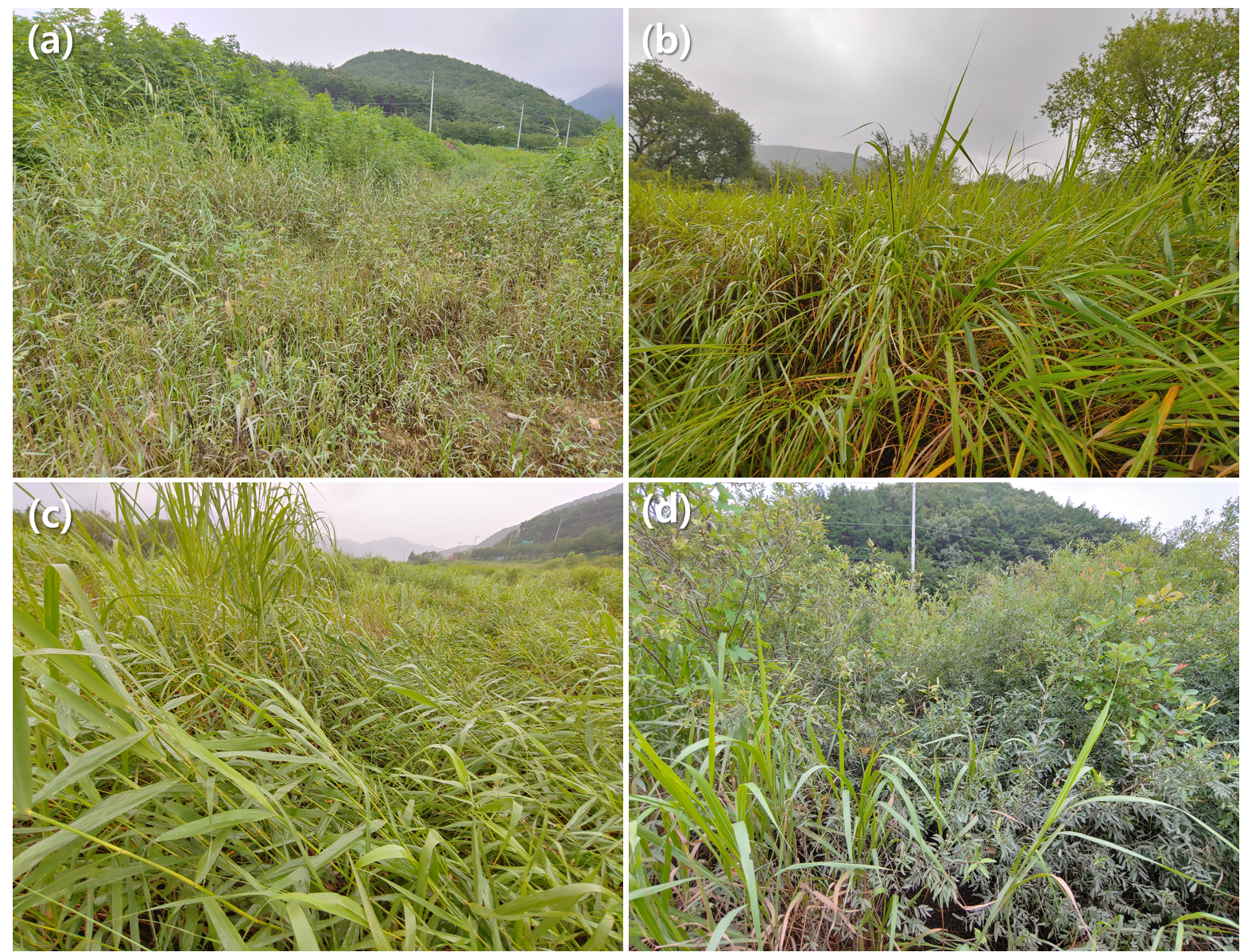

Supplement: Supplemental Information 1 — Photographs of each vegetation type, which were dominated by Setaria viridis (a), Miscanthus sacchariflorus (b), Phragmites communis (c), or Salix species (d). [file peerj-10-14050-s001.png]
