# Supplementary material for: Endangered plant species under differing anthropogenic interventions: how to preserve Pterygopleurum neurophyllum in Wondong wetland?
Source: PeerJ. 2022 Sep 28;10:e14050. doi: 10.7717/peerj.14050 (PMC9526420; doi:10.7717/peerj.14050)
Supplement: Supplemental Information 2 — Table S1. List of flora taxa detected in each vegetation type (A: Setaria type; B: Miscanthus type; C: Phragmites type; D: Salix type). Asterisk indicates exotic plant species. [file peerj-10-14050-s002.docx]

Table S1. List of flora taxa detected in each vegetation type (A: *Setaria* type; B: *Miscanthus* type; C: *Phragmites* type; D: *Salix* type). Asterisk indicates exotic plant species.

| Order | Family | Scientific name | Incidence in each vegetation type | | | |
| --- | --- | --- | --- | --- | --- | --- |
|  |  |  | A | B | C | D |
| Apiales | Apiaceae | *Pterygopleurum neurophyllum* (Maxim.) Kitag. |  | ○ |  |  |
| Asterales | Asteraceae | *Ambrosia artemisiifolia* L.* | ○ |  | ○ |  |
|  |  | *Ambrosia trifida* L.* | ○ | ○ |  |  |
|  |  | *Artemisia indica* Willd. | ○ | ○ |  |  |
|  |  | *Bidens bipinnata* L. | ○ |  |  |  |
|  |  | *Conyza canadensis* (L.) Cronquist* | ○ | ○ |  | ○ |
|  |  | *Conyza sumatrensis* (Retz.) E. Walker* |  | ○ |  |  |
|  |  | *Cosmos bipinnatus* Cav.* | ○ |  |  |  |
|  |  | *Eclipta prostrata* (L.) L. | ○ |  |  |  |
|  |  | *Xanthium orientale* L.* | ○ |  |  |  |
| Capparales | Brassicaceae | *Rorippa palustris* (L.) Besser | ○ |  |  |  |
| Caryophyllales | Amaranthaceae | *Achyranthes bidentata* var. *japonica* Miq. |  | ○ |  | ○ |
|  |  | *Amaranthus patulus* Bertol.* | ○ |  |  |  |
|  | Chenopodiaceae | *Chenopodium album* L.* | ○ |  |  |  |
| Commelinales | Commelinaceae | *Commelina communis* L. | ○ |  |  |  |
| Cyperales | Cyperaceae | *Carex dimorpholepis* Steud. |  | ○ | ○ | ○ |
|  |  | *Carex dispalata* Boott |  | ○ | ○ |  |
|  |  | *Carex neurocarpa* Maxim. | ○ | ○ |  |  |
|  |  | *Carex thunbergii* var. *appendiculata* (Trautv.) Ohwi |  | ○ |  | ○ |
|  |  | *Cyperus amuricus* Maxim. | ○ |  |  |  |
|  |  | *Scirpus radicans* Schkuhr |  |  | ○ |  |
|  | Poaceae | *Bromus japonicus* Thunb. |  | ○ |  |  |
|  |  | *Echinochloa crusgalli* (L.) P. Beauv. | ○ |  |  |  |
|  |  | *Hemarthria sibirica* (Gand.) Ohwi |  | ○ |  |  |
|  |  | *Phragmites australis* (Cav.) Trin. ex Steud. | ○ | ○ | ○ | ○ |
|  |  | *Zizania latifolia* (Griseb.) Turcz. ex Stapf |  | ○ | ○ |  |
|  |  | *Elymus tsukushiensis* Honda |  | ○ |  | ○ |

Table S1. Continued.

| Order | Family | Scientific name | Incidence in each vegetation type | | | |
| --- | --- | --- | --- | --- | --- | --- |
|  |  |  | A | B | C | D |
|  |  | *Miscanthus sacchariflorus* (Maxim.) Hack. |  | ○ | ○ | ○ |
|  |  | *Setaria viridis* (L.) P. Beauv. | ○ |  |  |  |
| Euphorbiales | Euphorbiaceae | *Acalypha australis* L. | ○ | ○ |  |  |
|  |  | *Phyllanthus ussuriensis* Rupr. & Maxim. | ○ |  |  |  |
| Fabales | Fabaceae | *Amorpha fruticosa* L.* |  |  |  | ○ |
|  |  | *Glycine soja* Siebold & Zucc. |  | ○ |  |  |
|  |  | *Kummerowia striata* (Thunb.) Schindl. | ○ |  |  |  |
|  |  | *Lespedeza cuneata* (Dum. Cours.) G. Don. |  | ○ | ○ | ○ |
| Juncales | Juncaceae | *Juncus decipiens* (Buchenau) Nakai | ○ |  |  |  |
| Lamiales | Lamiaceae | *Mosla dianthera* (Buch.-Ham. ex Roxb.) Maxim. | ○ | ○ |  |  |
| Liliales | Dioscoreaceae | *Dioscorea japonica* Thunb. | ○ |  |  |  |
| Polygonales | Polygonaceae | *Persicaria hydropiper* (L.) Delarbre |  |  | ○ |  |
|  |  | *Persicaria lapathifolia* (L.) Delarbre |  | ○ | ○ | ○ |
|  |  | *Persicaria maackiana* (Regel) Nakai ex T. Mori | ○ |  | ○ |  |
|  |  | *Persicaria perfoliata* (L.) H. Gross | ○ | ○ | ○ | ○ |
|  |  | *Persicaria sagittata* var. *sieboldii* (Meisn.) Nakai |  | ○ |  |  |
|  |  | *Polygonum nodosum* Person | ○ | ○ |  |  |
|  |  | *Rumex nipponicus* Franch. & Sav.* | ○ |  | ○ |  |
| Primulales | Primulaceae | *Lysimachia davurica* Ledeb. |  | ○ |  |  |
| Rosales | Rosaceae | *Potentilla kleiniana* Wight & Arn. | ○ | ○ |  |  |
|  |  | *Rosa multiflora* Thunb. | ○ | ○ | ○ | ○ |
|  |  | *Rubus parvifolius* L. |  | ○ |  |  |
| Rubiales | Rubiaceae | *Galium spurium* L. |  |  | ○ |  |
|  |  | *Galium tokyoense* Makino |  | ○ | ○ |  |
|  |  | *Paederia foetida* L. |  | ○ | ○ | ○ |
|  | Salicaceae | *Salix chaenomeloides* Kimura |  |  |  | ○ |
|  |  | *Salix pierotii* Miq. |  | ○ |  | ○ |
|  |  | *Salix koriyanagi* Kimura ex Goerz | ○ |  | ○ | ○ |

Table S1. Continued.

| Order | Family | Scientific name | Incidence in each vegetation type | | | |
| --- | --- | --- | --- | --- | --- | --- |
|  |  |  | A | B | C | D |
| Sapindales | Aceraceae | *Acer tataricum* subsp. *ginnala* (Maxim.) Wesm. |  | ○ |  |  |
| Urticales | Cannabaceae | *Humulus japonicu*s Siebold & Zucc. | ○ | ○ | ○ | ○ |
|  | Ulmaceae | *Ulmus parvifolia* Jacq. |  |  |  | ○ |
| Violales | Cucurbitaceae | *Actinostemma lobatum* (Maxim.) Franch. & Sav. |  | ○ | ○ |  |
|  | Violaceae | *Viola lactiflora* Nakai |  |  |  | ○ |
|  |  | *Viola raddeana* Regel |  |  |  | ○ |
